# Engineered 3D-Printable Nanohydroxyapatite Biocomposites with Cold Plasma-Tailored Surface Features to Boost Osseointegration

**DOI:** 10.1021/acsami.4c22032

**Published:** 2025-04-14

**Authors:** Rosalind Sin Man Chan, Sang Jin Lee, Fang Wang, Tianyu Zhou, Ravi Kishan, Ho Cheung Shum, Weifa Yang, Yu-xiong Su, James Kit Hon Tsoi, Ashish D. Diwan, B. Gangadhara Prusty, Kiho Cho

**Affiliations:** † Division of Applied Oral Sciences and Community Dental Care, Faculty of Dentistry, The University of Hong Kong, Hong Kong SAR 999077, China; ‡ Department of Mechanical Engineering, The University of Hong Kong, Hong Kong SAR 999077, China; § Division of Oral and Maxillofacial Surgery, Faculty of Dentistry, The University of Hong Kong, Hong Kong SAR 999077, China; ∥ Advanced Biomedical Instrumentation Centre, Hong Kong Science Park, Shatin, New Territories, Hong Kong SAR 999077, China; ⊥ Spine Labs, St George and Sutherland Clinical School, University of New South Wales, Randwick 2052, NSW, Australia; # Spine Service, Department of Orthopaedic Surgery, St George and Sutherland Clinical School, University of New South Wales, Kogarah 2217, NSW, Australia; ∇ School of Mechanical and Manufacturing Engineering, University of New South Wales, Sydney 2052, NSW, Australia; ○ ARC Centre for Automated Manufacture of Advanced Composites, School of Mechanical and Manufacturing Engineering, University of New South Wales, Sydney 2052, NSW, Australia

**Keywords:** resin composite, 3D printing, implant, orthopedic, bone reconstruction, cold plasma, nanohydroxyapatite

## Abstract

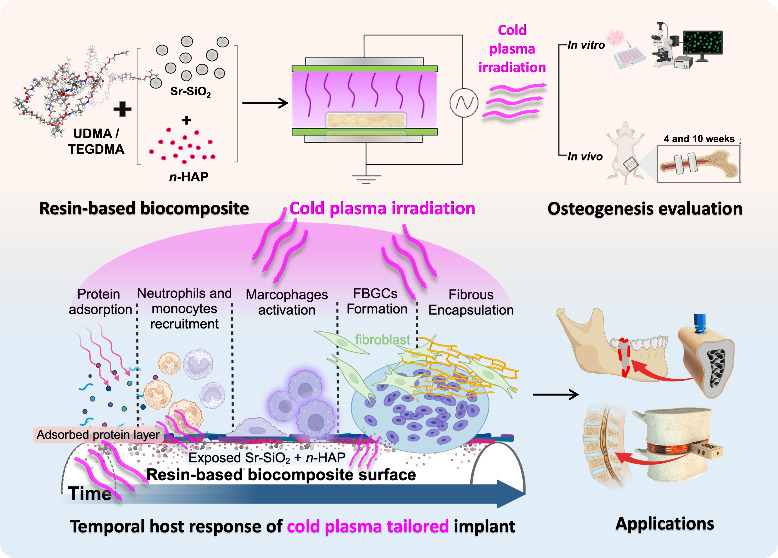

Medical implants, being biomaterials with increasing global use, continue to attract researchers focused on enhancing clinical performance. In situations requiring bone substitutes, there is a search for advancements in synthetic graft biomaterials, with polymer-based implants being one of the potential materials. Thus, this study aims to develop versatile nanohydroxyapatite (*n*HAP) biocomposites that can not only be generalized by resin composite systems but also be applicable for 3D printing, overcoming the limitations associated with traditional implants. Polymeric biocomposites are prepared by incorporating *n*HAPs and strontium-doped SiO_2_ glass particles (GPs) into a photocurable methacrylate monomer system, followed by 3 min of cold atmosphere plasma irradiation. In light of our findings, this medical implant possesses strong mechanical strength. Its surface hydrophilicity is enhanced through cold plasma treatment, which involves surface dry etching with nanoscale precision and exposing the embedded nanofillers to the outmost surface. This cold plasma treatment also induces osteogenic activity *in vitro* and bone integration *in vivo*. Furthermore, the 3D printability is demonstrated through the fabrication of a gyroid lattice structure. Collectively, this *n*HAP-biocomposite exhibits promising biomechanical and biological properties, providing potential for revolutionizing future implant applications in dental and maxillofacial reconstruction as well as orthopedic interbody fusion.

## 1 Introduction

In clinical practice, titanium (or alloy), ceramics, polyetheretherketone (PEEK), etc., serve as prevalent bone substitute biomaterials.^1^ Titanium (or alloy), although known for its high strength and biocompatibility, can cause stress shielding due to its high elastic modulus, which mismatches with natural bone.^2^ Although ceramics exhibit good chemical stability and a comparatively low occurrence of adverse biological reactions, they can be weakened or degraded when the pH level rises.^3,4^ PEEK possesses desirable aesthetic and mechanical properties and superior chemical resistance. However, it exhibits low surface energy and hydrophobic surfaces, rendering it inert to biological substances.^5^ Consequently, suitable osteoinductive biomaterials should be continuously considered with the specific constraints and demands for bone reconstruction.

In order to address these constraints, ongoing research efforts are concentrated on the development of novel biomaterials along with manufacturing techniques. Polymer-based materials like PEEK, poly(methyl methacrylate) (PMMA), and bisphenol A diglycidyl methacrylate-triethylene glycol dimethacrylate (BisGMA-TEGDMA) copolymer composites are emerging as promising alternatives due to their biocompatibility.^6,7^ These materials have been found to be noncytotoxic yet inert materials, indicating that they do not elicit unfavorable reactions or tissue damage upon contact but also lack osteoconductive properties.^8,9^ The incorporation of fibers, such as metal (or oxide), bioactive glass,^10^ and hydroxyapatite,^11^ has been explored to enhance their mechanical properties and osteogenic capabilities.^12^ Nevertheless, PEEK and part of the polymers do not facilitate osseointegration without any surface modifications. Instead, only mechanical interlockings are established at the interface between PEEK and bone.^13^

Surface modification is vital, as it directly influences the surface biocompatibility and the subsequent osteogenesis,^14^ especially when polymeric surfaces exhibit a relatively low wettability.^15^ The wetting behavior of a surface is influenced by both its physical features, including surface roughness, and its chemical qualities, such as heterogeneity.^15^ By increasing the hydrophilicity of a surface, it is possible to promote cell attachment^16^ while also preventing protein fouling, which can lead to non-biocompatibility.^17^ Cold atmosphere plasma (CAP) is a potential considerable surface modification method for orthopedic implants, which affects the physical, chemical, and biological properties of material surfaces^18^ without changing their bulk properties. This is crucial when working with 3D printing materials, where maintaining the mechanical integrity of the material is crucial. CAP uses atmospheric gases to convert inert surfaces into chemically active regions without raising temperatures. High-voltage ionized gases produce reactive oxygen species (ROS) that break C–C and C–H bonds, facilitating the formation of O–H bonds that are favorable for hydrophilicity, thereby improving the surface energy and wettability of the material.^19,20^ The effectiveness of CAP is influenced by process factors such as gas mixture, power supply, mode and duration of exposure, and inherent target cell or material characteristics. When oxygen (O_2_) is combined with helium (He), it can reduce the emission rates of He molecules and increase the concentration of reactive oxygen species (ROS).^21^ These chemical enhancement characteristics are difficult to obtain by using other approaches like chemical or thermal treatments that require harsher conditions or cause unwanted changes in the material structure. In contrast with chemical surface treatment, CAP is nontoxic and a dry procedure that has no detrimental impact on normal cells. It is therefore more environmentally friendly and suitable for use in biomaterials applications.^22^ Nevertheless, there is presently inadequate evidence to support the effectiveness of using polymer materials with plasma for improving surface biocompatibility.

In addition to improving surface biocompatibility, structural biocompatibility entails the optimal adaptation of the mechanical behavior of the host tissue, such as elastic modulus and strength. With 3D printing technology, the fabrication of complex structures with precise control over composition and geometry becomes possible. Incorporating biomimetic, bioinspired architectures such as honeycomb or re-entrant structures has been shown to significantly improve the strength, stiffness, and impact resistance of implants.^23^ Additionally, cellular geometries, such as bone-like structures with re-entrant, hybrid concave–convex cell geometries, have shown superior performance in terms of lightweight design and energy absorption capacity.^24^ These advancements provide improved functionality, better integration, and enhanced patient outcomes in the field of orthopedics. The ideal interaction between biomaterials and host tissues occurs when both surface and structural biocompatibility are achieved.

**Figure 1:**
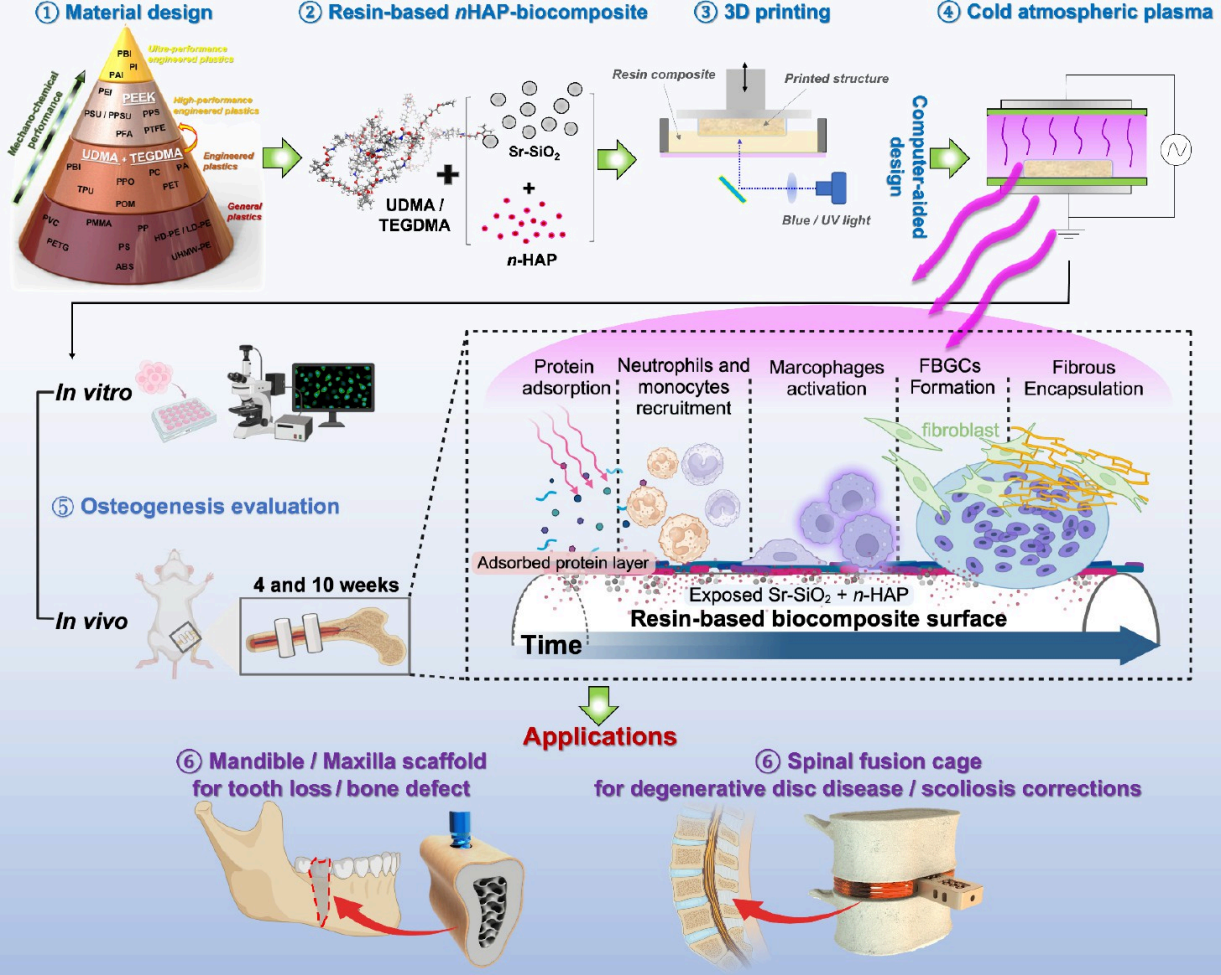
Schematic diagram of the 3D-printed and surface nanofeatured medical implants for bone reconstruction. Flowchart showing the process: ① Materials selection and design, ② fabrication of resin-based *n*HAP-biocomposites, ③ photocuring 3D printing, ④ post-surface modification using cold atmospheric plasma, ⑤ osteogenesis evaluation and schematic illustration of the bone integration hypothesis (temporal representation of the host response to fibrous encapsulation of an implanted polymer material begins immediately upon contact with host fluids (e.g., blood, lymph, wound fluids)),^25^ and ⑥ application of medical implants to smart fusion cages for spinal surgery and defect-specific scaffolds for oral and maxillofacial reconstruction.

To this end, we present innovative multifunctional hierarchically structured biocomposite resins by employing 3D printing technologies (Figure 1). In this study, we initially employ CAP technology to transform a biotolerant resin composite surface into a bioactive one, systematically showing as a proof of concept that the nanohydroxyapatite (*n*HAP) biocomposite is capable of being activated via cold atmospheric plasma and amenable to 3D printing, offering cost-effectiveness, exceptional biocompatibility, and osteogenic characteristics while keeping the ability to mimic the internal porous cancellous bone structure.

## 2 Experimental Section

### 2.1 *n*HAP-Biocomposites Fabrication

Novel *n*HAP-biocomposites were synthesized by mixing monomers and fillers. The monomer systems (Figure S1) were prepared by mixing urethane-dimethacrylate (UDMA) and triethylene glycol dimethacrylate (TEGDMA) (Esstech, Inc., USA) with a mixing ratio of 80:20 wt %, and camphorquinone (CQ) (Esstech, Inc., USA), dimethylaminoethyl methacrylate (DMAEMA) (Sigma-Aldrich, USA), and diphenyliodonium hexafluorophosphate (DPI) (Shanghai Aladdin Biochemical Technology Co., Ltd., China) were mixed in a ratio totalling 1.0 wt % for initiator and co-initiators. This monomer system was continuously stirred overnight using a magnetic stirrer with a speed of 250 rpm. Then, 10 and 20 wt % of strontium-doped SiO_2_ glass particles (GPs, mean diameter: 700 nm) (Cera Dynamics Ltd., England) were respectively added to the prepared monomer system and mixed using a high-speed turbulent mixer (ARE-310, THINKY mixer, Japan) 3 times at 2000 rpm, each cycle lasting 20 s. An additional 2 wt % of nanohydroxyapatite (*n*HAP, <200 nm powder, Sigma-Aldrich, USA) was mixed using the same process. Prior to the 3D printing process, a vacuum at −95.0 kPa was applied for 5 min to fully eliminate air bubbles in the composites.

### 2.2 Specimen Preparation

Cylindrical specimens were fabricated using a stereolithography 3D printer (M-one, MakeX Ltd., China) for mechanical testing. Teflon molding was employed for wettability testing and *in vitro* and *in vivo* experiments due to the small dimensions of the implants. Pure resin (PR) was set as a control, while PEEK was employed as the comparison group. Groupings were named according to filler additives: G10 (10 wt % GPs), H2@G10 (*n*HAP with 10 wt % GPs), and H2@G20 (*n*HAP with 20 wt % GPs). For *in vitro* study, resin composite disks were produced using a Teflon mold that was 6 mm in diameter and 4 mm deep. A cover glass was used to fabricate a smooth surface, and light curing was achieved by an LED light curing unit (intensity: 1200 mW/cm^2^) (Bluephase Style, Ivoclar Vivadent, Liechtenstein), standardized 60 s for top and bottom side. After photopolymerization, the edges were carefully trimmed by abrasion paper. All samples were then subjected to ultrasound and air-dried before use.

### 2.3 3D Printability Evaluation

A digital light processing (DLP) 3D printer installed with a visible light injector containing blue light (λ ≈ 450 nm) was used to print mechanical compression test specimens and to test printability. All specimens in the shape of circular columns (10.0 × 7.0 mm, diameter × height) were printed out vertically with a 50 μm layer thickness and a 3.5 s exposure time, then post-irradiated by 60 mW/cm^2^@450 nm light for another 1 h on a rotating stage. The surfaces of the postcured specimens were polished using 1500 mesh abrasion paper prior to the mechanical test.

### 2.4 Mechanical Properties

The compression tests were performed with a universal testing machine (Instron 3369, Instron Ltd., USA) with a 1 kN load cell, and a compression force was applied to the specimens in displacement control mode with a 1 mm/min loading speed. The collapse strength and compression modulus of the 3D-printed specimens were obtained from the stress–strain data.

### 2.5 Cold Atmosphere Plasma Irradiation and Characterization

A dielectric barrier discharge (DBD) system (CTP-2000K, Corona Lab, China) was used for the CAP treatment (Figure 3a). The CAP apparatus comprised a gas inlet and a chamber for specimen positioning. Helium and oxygen (purity 99.8%) gas flows (4.0 L/min, 100.0 mL/min) were mixed immediately after being discharged and maintained by the voltage and current at 70 kV and 2.0 A, respectively. The mixture of He and O_2_ was selected because it provides a conducive environment for generating reactive oxygen species (ROS),^26^ and voltage settings were chosen based on previous studies^22,27^ in generating high-energy ionized species that can modify polymer surfaces without causing a thermal distortion current. Plasma gas was introduced into the chamber from the above inlet and exposed to the sample surfaces for different times. The samples were stochastically collected to examine the surface using SEM (SU1510, Hitachi, Japan). The elemental releases of calcium (Ca) and phosphorus (P) in *n*HAP-biocomposites immersed in HBSS solutions were measured at immersion times of 1, 7, 14, and 30 days using inductively coupled plasma-optical emission spectrometry (ICP-OES) (Spectro Arcos, SPECTRO Analytical Instruments GmbH, Germany). The concentration of each sample was determined using the standard curves.

The irradiation time was optimized based on the observed effects on surface roughness and hydrophilicity.^28^ The measurements of water contact angle were carried out by an optical tensiometer (Theta Flow, Biolin Scientific, Sweden). Surface roughness and CAP etching thickness of *n*HAP-biocomposites over time were examined using AFM (Dimension Edge, Bruker, Germany). Through mechanical testing, the material group (H2@G20) with the best mechanical performance was selected for the subsequent biology experiments. They were divided into two subgroups: one exposed to plasma irradiation (H2@G20-P) and the other without irradiation (H2@G20), while tissue culture polystyrene plastic (TCPs) served as a positive control.

### 2.6 Cell Culture

Murine preosteoblast cell line MC3T3-E1 cells (ATCC, USA) were cultured in minimal essential medium α (MEM α; Thermo Fisher Scientific, USA) supplemented with 10% fetal bovine serum (FBS; Thermo Fisher Scientific, USA), 100 U/mL penicillin, and 100 μg/mL streptomycin (Thermo Fisher Scientific, USA) at 37 °C in a humidified atmosphere with 5% CO_2_. Cells were harvested from a monolayer culture by using 0.25% trypsin-EDTA (Thermo Fisher Scientific, USA) with a centrifuge and resuspended in fresh medium to the desired concentration. Sterilization was performed in an autoclave for 15 min at 121 °C. The *in vitro* osteogenic activity tests were conducted in triplicate.

### 2.7 Cell Live/Dead Viability Evaluation

To investigate the effect of the CAP-treated surface on the viability of MC3T3-E1, fluorescence staining with Calcein-AM in green for viable cells and propidium iodide (PI) in red for dead cells (C2015L, Beyotime, China) was analyzed. MC3T3-E1 cells were seeded on each sample disk at a density of 1 × 10^5^ cells/mL with 300 μL MEM α. Three fields of view for observation were randomly selected, and the percentage of live/dead cell numbers was calculated for 1, 3, and 5 days of incubation by ImageJ v1.53u (National Institutes of Health, USA).

### 2.8 *In Vitro* Cell Adhesion and Proliferation

Cell adhesion and proliferation were quantified using a Cell Counting Kit-8 (CCK-8, C0038, Beyotime, China). Cell suspensions with a density of 1 × 10^5^ cells/mL were seeded on *n*HAP-biocomposites in a 48-well plate, 300 μL for each well. The culture was terminated at 3 and 6 h for cell adhesion evaluation and 1, 3, and 5 days after cultivating at the designated time. CCK-8 working solution was then added to each well and incubated for 1 h. Then, 100 μL of the supernatant was taken from each well plate, and absorbance was measured at a 450 nm wavelength. The cell morphology was subsequently viewed via SEM.

### 2.9 Osteogenic Activity of Differentiated MC3T3-E1

For cytoskeleton observation, the cells were first fixed with 4% paraformaldehyde (PFA) for 10 min, then permeabilized with 0.1% Triton X-100 in phosphate-buffered saline (PBS) for another 10 min. Cells were incubated in the diluted (1/100) recombinant anti-vinculin primary antibody (ab129002, abcam, UK) in 1% bovine serum albumin (BSA) (9998S, Cell Signaling Technology, USA) in PBST (PBS + 0.1% Tween 20) overnight at 4 °C. After, the solution was decanted, followed by three washes with PBS. Then, goat anti-rabbit secondary antibody Alexa Fluor 488 (A-11008, Thermo Fisher Scientific, USA) was applied to label focal adhesions for 1 h at room temperature. The F-actin cytoskeleton was visualized using diluted Rhodamine Phalloidin (R415, Thermo Fisher Scientific, USA) according to the manual and incubated for 40 min, followed by nuclei staining for 10 min using diamidino-2-phenylindole (DAPI) (62248, Thermo Fisher Scientific, USA). Finally, all fluorescent images (20×) were captured with a ZEISS confocal laser scanning microscope (CLSM) (LSM 900, ZEISS, Germany) with appropriate filter sets. The average mean vinculin fluorescence intensity, average cell area, and line scan for vinculin quantification along the cell plane were calculated via ImageJ.

For analyzing the osteogenic differentiation process with or without plasma irradiation, the MC3T3-E1 cells were grown in the osteogenic induction medium (OIM), which was additionally supplemented with 50 μg/mL vitamin C, 0.01 M β-glycerophosphate, and 10^–7^ M dexamethasone, which was changed every 2–3 days after the cells reached ∼80% of confluency; 1 × 10^5^ cells/mL was seeded on the samples. The alkaline phosphatase (ALP) expression was assayed by an ALP activity kit (ab83369, abcam, UK), and the total amount of protein was normalized using a BCA protein assay kit (23227, Thermo Fisher Scientific, USA). After incubation for 7 and 14 days, the cells were lysed, and the supernatants were collected for microplate reading according to the manual.

Alizarin red staining (ARS) (A5533, Sigma-Aldrich, USA) was used to detect cell mineralization for 21 days. Cells were grown on the samples in OIM at a density of 1 × 10^5^ cells/mL. Induced cells were rinsed with PBS before 20 min of fixation in 4% paraformaldehyde (PFA). Subsequently, filtered ARS solution at 40 mM, pH 4.2 was added and stained for 5 min at room temperature. After observation of calcium nodules under a stereomicroscope (SMZ18, Nikon, Japan), semiquantification was performed by dissolving the calcified deposits with 10% cetylpyridinium chloride (C9002, Sigma-Aldrich, USA), and the absorbance was measured at 562 nm.

### 2.10 Experimental Grouping of Rats

The present study utilized a total of 28 male CD(SD)IGS (Sprague–Dawley) rats, aged 10–12 weeks and weighing 400–500 g. The study was conducted following review and approval from the HKU Committee on the Use of Live Animals in Teaching and Research (CULATR #22-280). The study was designed to include two groups, with 7 rats assigned to each group, and two end points at 4 and 10 weeks. Three rats were dedicated to the push-out test, while four rats were assigned to histology and nanohardness analysis.

### 2.11 *In Vivo* Osteointegration Appraisal

To create the implants, biocomposites were injected into an assemblable Teflon mold (Figure S2) and light cured for 2 min before polymerization into screw-shaped implants (*D* = 2.20 ± 0.05 mm, *L* = 4 ± 0.05 mm) (Figure 6b). Details of the surgery procedure can be found in the Supplementary Animal Experimental Section. After a two week recovery period from surgery, the position of the implant was determined using dual-energy X-ray absorptiometry (UltraFocusDXA, Faxitron, USA). Following this, the rats underwent *in vivo* micro-CT scans (SkyScan1276 machine, Bruker, Germany) at 4, 6, 8, and 10 weeks after anesthesia, with each scan taking approximately 9 min per sample. The scan parameters included a voltage of 70 kV, a current of 200 μA, an exposure time of 1000 ms, and a rotation step of 0.400°. Image reconstruction and visualization were performed using NRecon v2.0.0.5 software (Bruker, Kontich, Germany), which applied 30% beam hardening reduction and 2-ring artifact corrections. The region of interest (ROI) was set as the middle bulge part of the implant-cancellous area, measuring 1.0 mm in length and 2.4 mm in diameter. CTAn v1.23.0.2 software was used to quantify parameters such as trabecular bone volume to total volume fraction (BV/TV), trabecular thickness (mm), trabecular number (Tb. N), and bone mineral density (BMD). Additionally, CTVol v2.3.2.0 software was employed to create 3D models.

### 2.12 Bone-Implant Push-Out Strength

To evaluate the *in vivo* strength of bone-implant fixation, fresh rat femurs were extracted post-sacrifice and kept moist. The rat femur and the implant were mounted on a fixture in a perpendicular axial position. A loading rod with a diameter of 1.6 mm (Figure 5b) exerted a push-out force on the implant at a speed of 1 mm/min. The maximum push-out (PO) strength (N) was determined from the load–displacement curves, and the osteointegration area between the implant and bone was calculated using the formula
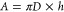
1Here, *A* represents the osteointegration area in mm^2^, *D* is the diameter of the implant body in mm, and *h* is the height of the implant in mm.

Afterward, the push-out strength (MPa) was calculated as
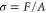
2σ represents the fixation strength in MPa, and *F* is the maximum load at failure in N. Stress (MPa) was determined by dividing the push-out load by the osteointegration area, and a stress–displacement curve was presented. A video was recorded for the PO test process in Video S1.

### 2.13 Histological Evaluation

After fixation in 4% PFA for 1 week, femurs were sequentially dehydrated in ethanol, followed by infiltration in methyl methacrylate (MMA) (Technovit 9100, Kulzer, Germany) and being stored in a −15 °C fridge for polymerization. Undecalcified sections were cut into coronal sections and ground to #2000 (Ecomet 5, Buehler, USA) with a thickness of 100–130 μm for histology staining and nanohardness assessments. The presence of newly formed bone was observed using hematoxylin and eosin (H&E) staining and photographed with light microscopes (SMZ18, Nikon, Japan). The bone-implant contact ratio (BIC) was determined by measuring the extent of new bone in contact with the implant body, excluding disconnected areas. Toluidine blue combined with alizarin red staining was employed to check the collagen fibers and differentiate between new and old bone tissue via a polarizing microscope (LV100POL, Nikon, Japan). To evaluate the mineralization of newly formed bone, the nanohardness (MPa) of cortical and new cortical bone was examined using an AFM with a force of 40 μN using tapping mode.

### 2.14 Statistical Analysis

The Shapiro–Wilk test was used to check normality; one-way ANOVA followed by an LSD post-hoc test and nonparametric Kruskal–Wallis were performed to assess the difference among groups, correspondingly (*n* = 4). SPSS (IBM SPSS software v23.0, IBM, USA) was used for the statistical analysis, in which *p* < 0.05 was inferred as statistically significant (*p < 0.05, **p < 0.01, ***p < 0.001, ****p < 0.0001).

## 3 Results and Discussion

### 3.1 3D Printability and Mechanical Properties

**Figure 2:**
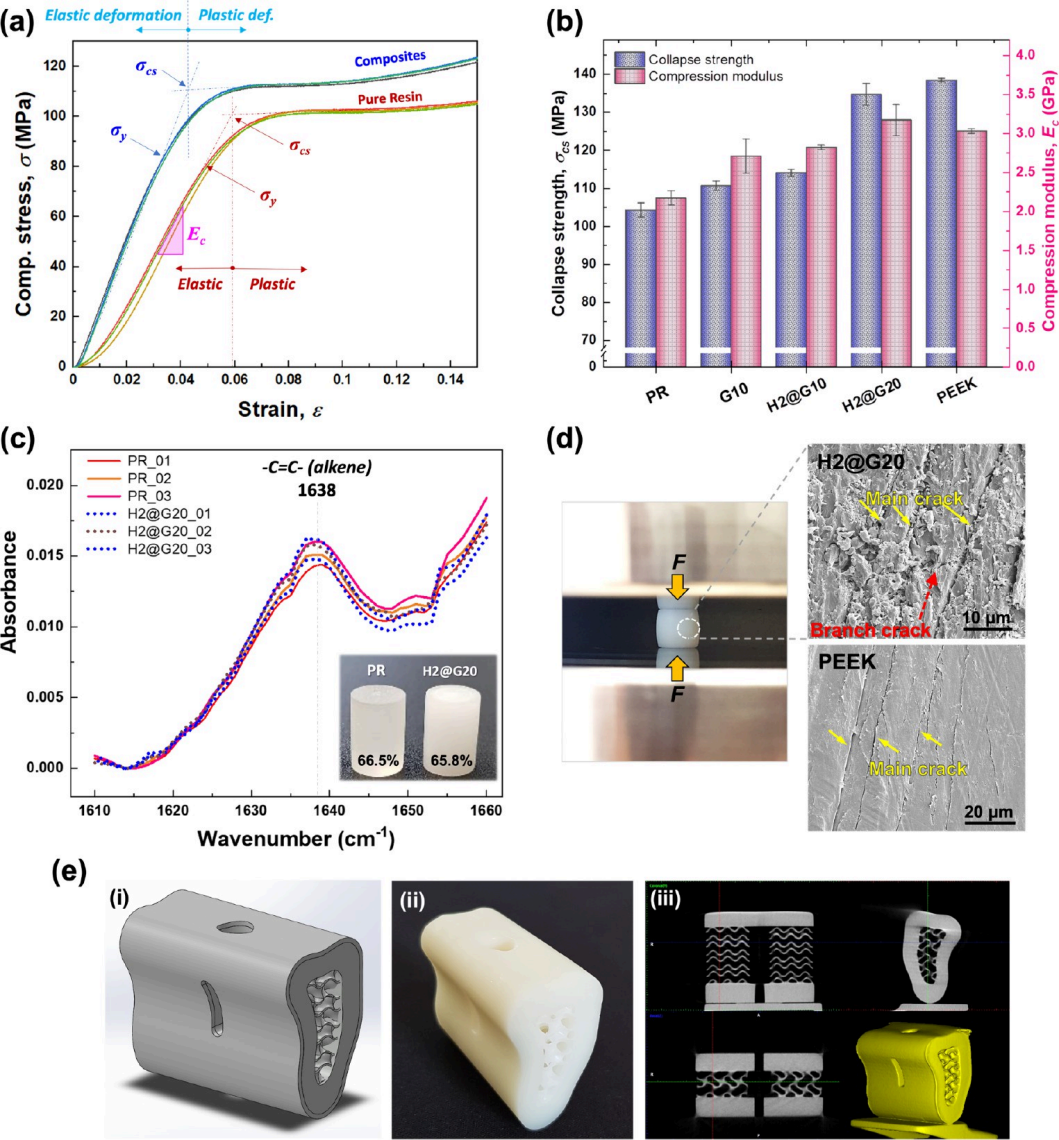
Mechanical characterization of *n*HAP-biocomposites. (a) Collapse strength (σ_*cs*_) and compression modulus (*E*_*c*_) are calculated from the stress–strain curves of compression tests. (b) Comparative analysis of collapse strength and compression modulus of the biocomposites against those of pure resin and PEEK. (c) The FTIR spectra around the 1638 cm^–1^ peak after post-photocuring pure resin and H2@G20, indicating no significant difference in the degree of conversion between the two groups. (d) SEM images of the fractured surface of H2@G20 (top) and PEEK (bottom) after compression test (left). The presence of a branch crack (red arrow) between the main crack slip lines (yellow arrow) in H2@G20 indicates an increase in crack propagation resistance. (e) A partial mandibular scaffold is designed using 3D CAD (i) and 3D-printed (ii). The internal structure is examined through CBCT analysis (iii).

The filler fraction plays a crucial role in optimizing 3D-printable biocomposites, as it affects both the flowability (viscoelasticity) of the composite during the 3D printing process and the mechanical properties of the composite after polymerization.^29^ Strontium (Sr) has been widely used to improve the radiopacity of various dental materials, exhibiting mild antimicrobial properties and bone regeneration effects.^30^ In this study, Sr-doped SiO_2_ was utilized to enhance the quality of radiographical images. The effects of varying weight fractions of GPs, ranging up to 20 wt %, on the collapse strength (σ_*cs*_) and elastic modulus (*E*_*c*_) in compression tests were investigated (Figure 2a,b). Figure 2a provides a description of the concepts of collapse strength and elastic modulus, as derived from the stress–strain curves obtained during the compression tests. The collapse strength and compression modulus are key parameters for evaluating biomaterials under load-bearing conditions. Collapse strength describes the capability to resist external loads without permanent deformation, ensuring an intact structure, while compression modulus indicates the stiffness of a biomaterial, which is quite important in providing adequate support to the bone tissue during healing and regeneration. The influence of incorporating 2 wt % of *n*HAPs^31,32^ was examined and finally compared with PEEK, which is widely utilized in dental and orthopedic surgeries. Composites reinforced with 10 wt % GPs (G10) exhibited improved collapse strength (110.63 ± 1.19 MPa) and compression modulus (2.71 ± 0.22 GPa) compared to the values of the pure resin (PR) system, 104.25 ± 1.89 MPa and 2.17 ± 0.09 GPa, respectively. However, the addition of 2 wt % *n*HAPs to GP10 (H2@G10) did not significantly contribute to the further increase in strength and modulus. The optimized filler composition, consisting of 20 wt % GPs and 2 wt % *n*HAPs (H2@G20), resulted in increased collapse strength (134.67 ± 2.85 MPa) and modulus (3.17 ± 0.20 GPa), which were comparable to the properties of PEEK. Lin et al. developed a bone-mimicking 3D printing resin using a high content of ceramic fillers; its Young’s modulus reached the value of real human bone, yet its biological properties have not been further verified.^33^

The degree of conversion (DC) was found to not be significantly different between the PR and the H2@G20 group after a post-photocuring process. The DC values were 66.5 ± 1.1% and 65.8 ± 0.6% (Figure 2c), respectively. This similarity can be attributed to both materials being fully photocured to the inner core for 1 h under high-intensity blue light. GPs block and change the direction of crack propagation, and the applied compressive force and energy can be efficiently absorbed and transmitted through the well-distributed fillers (Figure 2d), especially increasing the mechanical fracture strength.^34^ To evaluate the 3D printability of the H2@G20 group, an artificially designed partial mandible was printed (Figure 2e). The gyroid lattice structure acquired through 3D printing was used to mimic the internal porous cancellous bone architecture. This structure is more open in terms of density and has a larger surface area, thus having the capability to offer beneficial mechanical support and strength.^35^ Consequently, the structure enhances the load transfer capacity, mitigates stress concentration, and enhances compression resistance.^36^ Moreover, the larger surface space within the area and small pores permit more cell attachment.^37^ High-resolution cone beam computed tomography (CBCT) imaging enables precise visualization of the internal architecture of the biocomposite, which is essential for assessing the structural integrity of 3D-printed medical implants.^38^ The design of the mandible uses a gyroid lattice structure with a wall thickness of 300 μm to mimic the internal porous cancellous bone structure. As shown in Figure 2e(iii), investigation through CBCT image analysis showed excellent 3D printing feasibility.

### 3.2 Cold Atmospheric Plasma Treatment and Surface Modification

**Figure 3:**
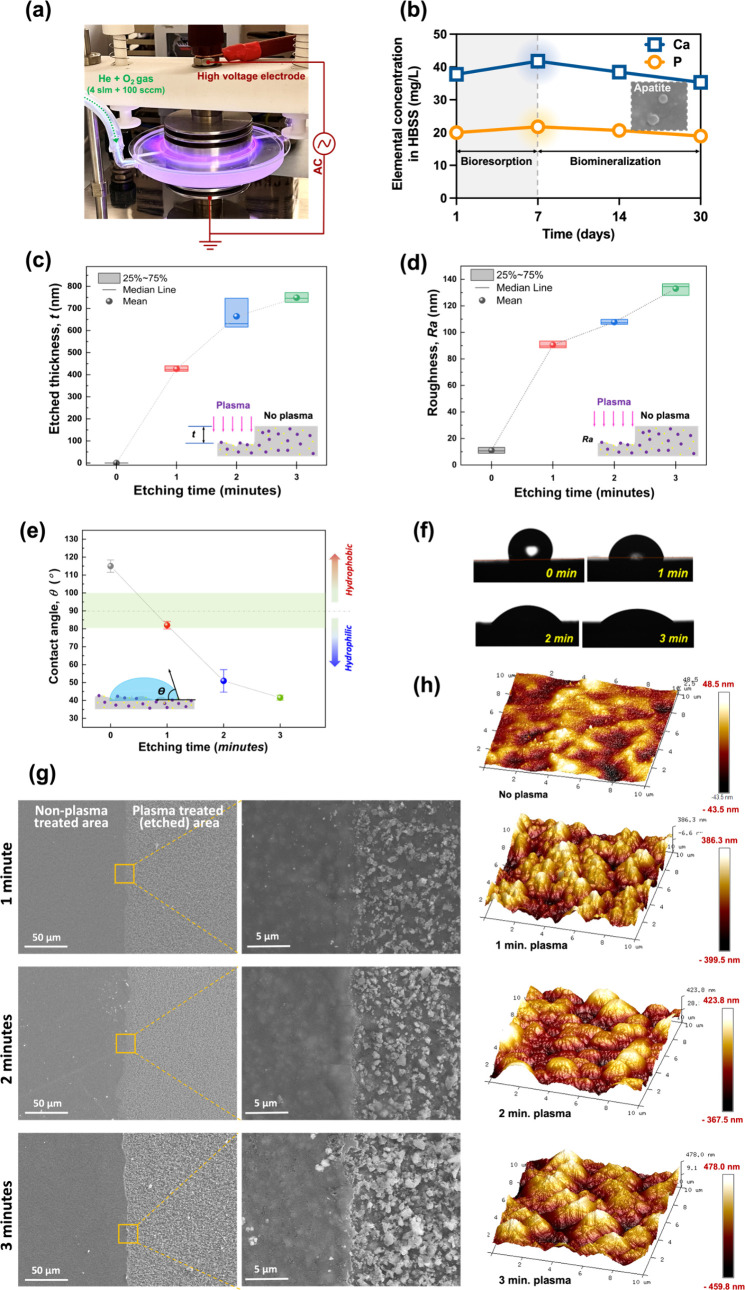
Post-surface modification on the *n*HAP-biocomposite surface (H2@G20) using CAP. (a) Set-up of high-voltage pulsed CAP with helium–oxygen feeding gas. (b) Ca and P elemental release after immersion in HBSS. (c) Etched thickness and (d) surface roughness obtained by AFM analysis, (e) static water contact angle measurement, and (f) lateral view images of water droplets on the biocomposite surface as a function of the CAP treatment time. (g) SEM and (h) AFM depictions of the biocomposite surface; the fillers in the CAP-treated area were easily exposed and visible in both visuals.

Surface properties such as biochemical composition, morphology, surface roughness, and wettability of medical implants play a crucial role in bone reconstruction.^39^ In the *n*HAP-biocomposite, bioactive fillers were embedded, and the composite had its outermost surface covered by the formed matrix even at the outermost micro/nanoscopic surface, which results in the inability of *n*HAPs to directly contact with cells and the outer environment. In order to expose the embedded nanofillers to the outer surface while altering the surface charged energy, CAP treatment was employed in this study (Figure 3a). This method was chosen because it operates at room temperature, preventing any thermal distortion of the polymer-based composite systems. As aforementioned, the H2@G20 group was selected based on its promising mechanical properties observed during preliminary tests without CAP treatment, while the H2@G20-P group was essentially the same as H2@G20 but was subjected to CAP.

After fillers were exposed, the release of calcium (Ca) and phosphorus (P) elements was observed to be increasing at 7 days and then progressively decreasing after 7 days (Figure 3b). Following immersion in HBSS for 7 days, newly formed spherical apatite particles were observed on the surface of *n*HAP-biocomposites. Concurrently, an initial high release of Ca was measured at 41.78 ± 0.39 mg/L, along with a P release of 21.76 ± 0.22 mg/L. This may be attributed to the active exchange and reaction between the ions of the resin and HBSS during the bioresorption period.^40^ By day 30, the release rates of both Ca and P decreased to 35.33 + 0.89 mg/L and 18.93 + 0.17 mg/L, respectively, which is typical of bone materials that release a burst of ions followed by a steady, lower level of release. It is suggested that during the biomineralization period, HBSS precipitated on the surface, resulting in the progressive formation of apatite and the remineralization of the *n*HAP-biocomposites.^40^ The release decreases slightly over time, suggesting that the material was reaching a point where its ion supply stabilizes. However, a sustained low-level release of Ca and P over several months is typically required to support ongoing mineralization and bone remodeling. Therefore, while this *n*HAP-biocomposite material is promising, further testing over a more extended period would help confirm its suitability for long-term mineralization.

Figure 3c depicts the relationship between the CAP etching time and the etching thickness of the *n*HAP-biocomposites. It revealed that as the etching time increased to 3 min, the outer surface of the specimens exhibited an increase in etched thickness, reaching an average value of 747.7 ± 18.1 nm. The helium–oxygen plasma treatment involved the utilization of activated oxygen radicals to break polymer bonds and chains,^41^ resulting in the gradual removal of the surface matrix at the nanoscale. Consequently, this process exposes embedded fillers such as GPs and *n*HAPs on the external surface, leading to an increase in surface average roughness (*R*_a_) with values rising from an initial 11.0 ± 1.8 to 133.0 ± 3.7 nm (Figure 3d) by plasma etching and oxidative reactions^42^ (Figure S3). Roughness modification is one of the measures that can improve wettability as surface roughness increases,^43^ thereby enhancing cell adhesion.^44^

In our study, plasma treatment has demonstrated the ability to decrease the water contact angle of *n*HAP-biocomposites from 78° to 35.5° (Figure 3e,f). Plasma-activated oxygen has a strong affinity to elements like Si, Ca, and P found in GPs and *n*HAPs (Figure S4). After 3 min of CAP treatment, a mild increase in Si, Ca, and P on the surface was observed, as well as a more uniform distribution of the elements, resulting in an increased concentration of oxygen on the exposed surfaces of these inorganic fillers. This enrichment of oxygen, along with the presence of oxygen-containing polar functional groups, leads to significant alterations in the surface characteristics of the composites. Specifically, as shown in Figure 3e,f, the water contact angle decreased from an average of 115.0° to 42.0° after a 3 min plasma treatment, indicating a transformation from a hydrophobic to a hydrophilic surface. An irradiation time of 3 min was most favorable for the *n*HAP-biocomposite, as increasing the exposure time beyond this did not provide additional benefits but risked overetching. Scanning electron microscopy (SEM) and atomic force microscopy (AFM) analyses, as depicted in Figure 3g,h, illustrate the alterations in surface topology of the CAP-treated composites for durations of 1, 2, and 3 min. These analyses reveal the exposure of nanofillers to the surface with a noticeable increase in surface roughness.

### 3.3 CAP Substrates Induce Cell Viability and Osteogenic Activity

**Figure 4:**
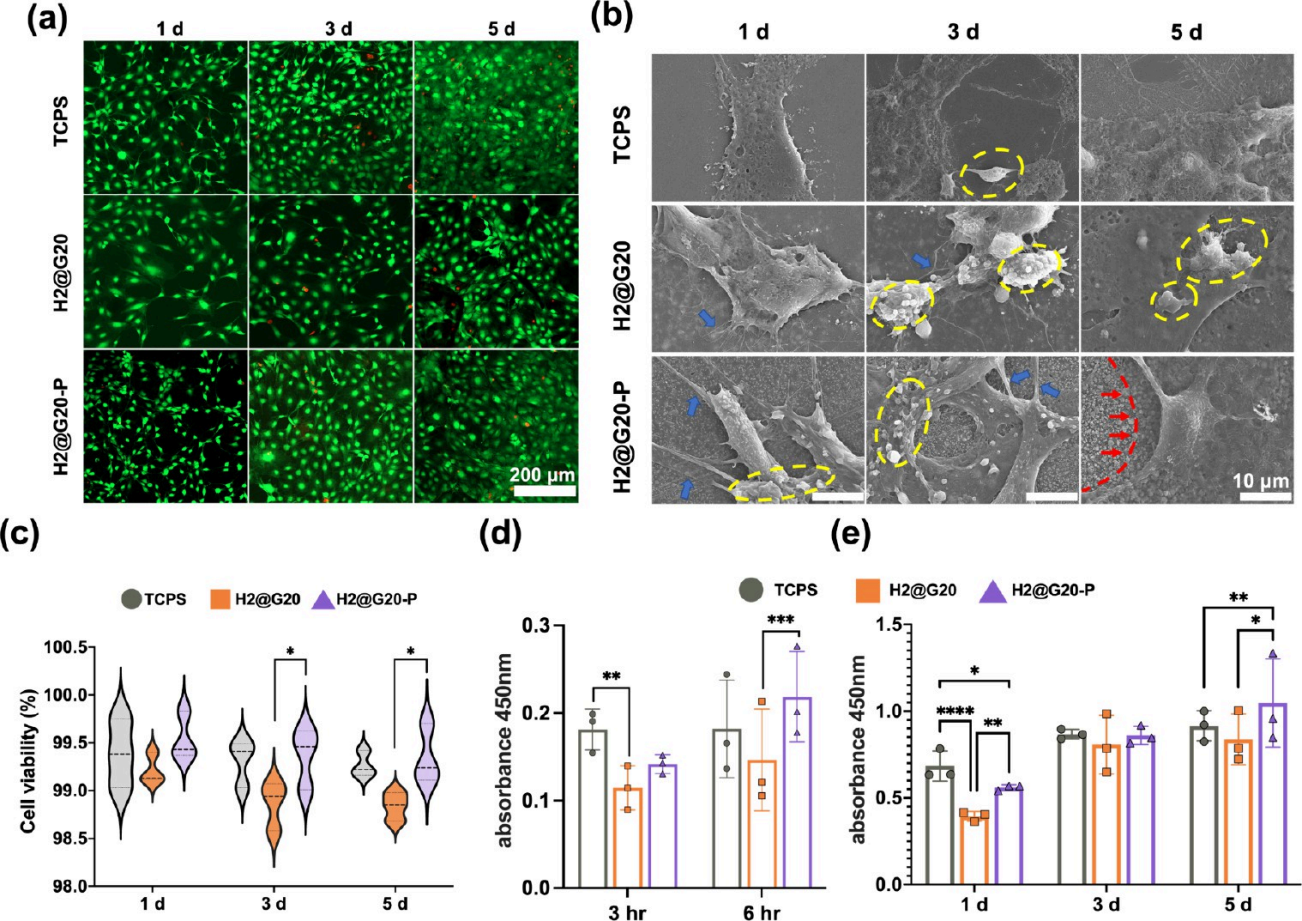
*In vitro* cell viability of MC3T3-E1. (a) Cell live/dead viability staining of MC3T3 cultured on the *n*HAP-biocomposite surfaces for 1, 3, and 5 days. Live cells (green fluorescence). Dead cells (red fluorescence). (b) Representative SEM images (×3000) of cell morphology: newly proliferating cells (yellow circles), thick pseudopods (blue arrow), and cell-secreted matrix covered the surface of the plasma etched material (red arrows). (c) Relative cell viability results quantified by live/dead cell staining. (d, e) OD value of cell adhesion and proliferation over time.

For polymeric biomaterials, the release of monomeric components was criticized for implicating cell viability; however, monomeric components were found to be noncytotoxic after complete polymerization. Different works found that UDMA had higher conversion than BisGMA when compared at similar diluent concentrations,^45^ and UDMA and TEGDMA were less cytotoxic compared to BisGMA.^46^ The 8:2 weight ratio of UDMA to TEGDMA was chosen based on our previous work.^47^ UDMA provides higher viscosity and strength, while TEGDMA offers better handling and helps improve polymerization. This ratio was optimized to balance cross-linking and flexibility. In the cell viability study (Figure 4a), the duration of culture time did not produce a statistical difference in cell viability, and it can be inferred that the composition of *n*HAP-biocomposite was biocompatible. Following 3 and 5 days of culture, the CAP-treated material (H2@G20-P) surface was found to have more viable cells (Figure 4c). These results indicated that the plasma could preserve cell viability without subsequent toxicity. Takamatsu et al. revealed that the use of helium CAP resulted in the production of nitric oxide (NO) radicals. This process involves the interaction of reactive oxygen and nitrogen species in the environment,^48^ wherein the NO radicals have been demonstrated to be beneficial for cell activity.^49^

The results of the 3 and 6 h cell adhesion to each group of materials are shown in Figure 4d. The H2@G20-P group showed a greater proportion at 6 h compared to the H2@G20 group (*p* < 0.001). By comparing the proliferation results of the three groups on day 1 (Figure 4e), the TCPs group showed the greatest absorbance, followed by the H2@G20-P group. After 5 days of culture, the absorbance of the H2@G20-P was significantly higher than that of the other two groups, indicating the enhanced proliferation and differentiation potential of the H2@G20-P group in the later time points. This finding was consistent with the results observed in SEM images (Figure 4b): upon 1 day of culture on the material, cells in the three groups were mostly star-like or polygonal, with newly proliferating cells (in yellow circles) visible on their surfaces; thick pseudopods were visible in the stretched ends of the H2@G20-P, as indicated by the blue arrows. In 3 and 5 days, the cells were fully spread flat, and functional particles could be seen on the cell surface. The matrix deposition around the cells increases and undergoes mineralization over time. By day 5, the cells in the H2@G20-P culture exhibited increased secretion of matrix, leading to being more distinct and visible (red arrows). As evidenced by this finding, CAP-treated *n*HAP-biocomposites can stimulate osteoblast proliferation.

**Figure 5:**
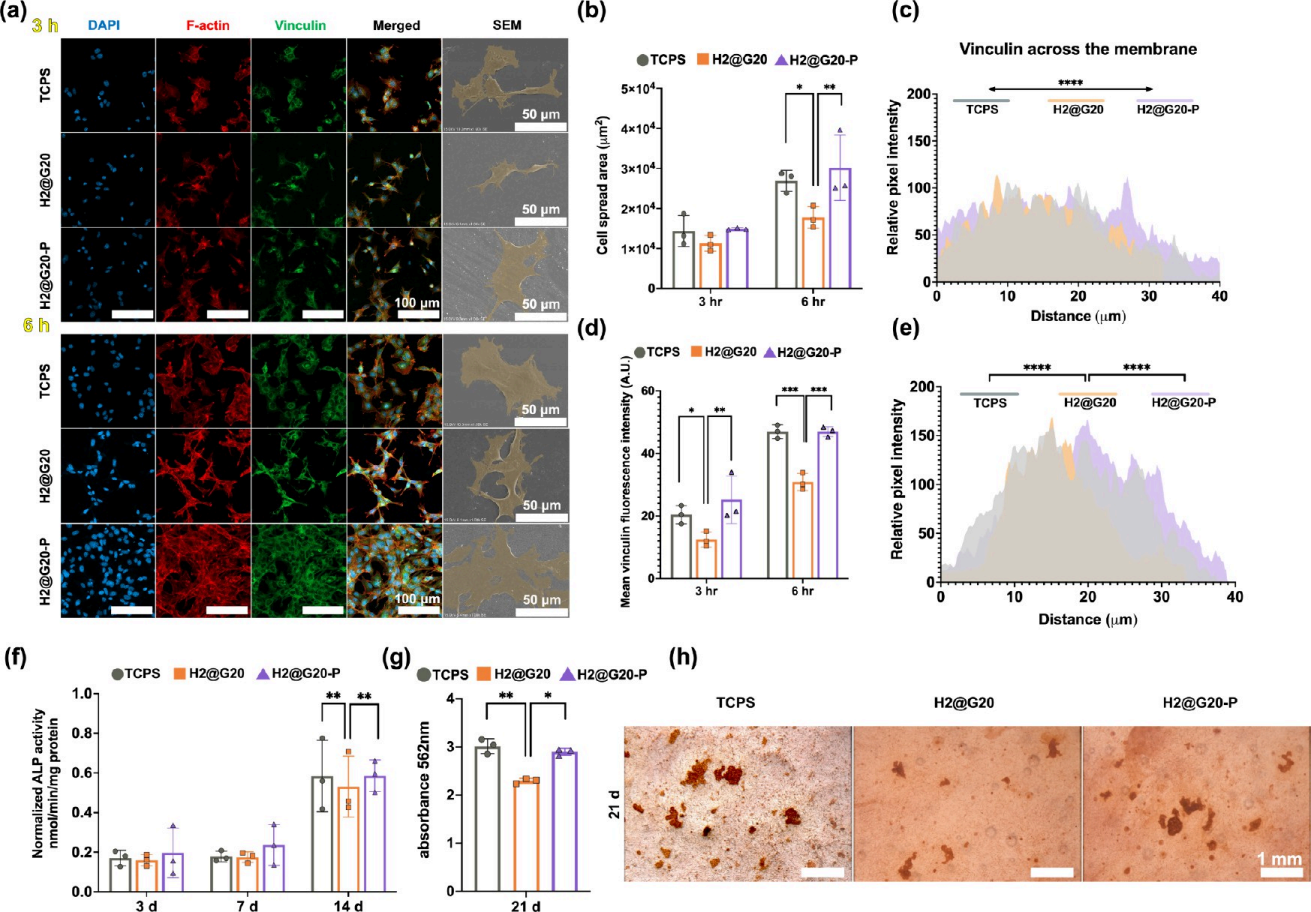
*In vitro* osteogenic activity of MC3T3-E1. (a) Cytoskeleton structure labeled by fluorescence and observed via CLSM: nuclei (blue, DAPI), F-actin cytoskeleton (red, Rhodamine Phalloidin), focal adhesions (green, goat anti-rabbit secondary antibody), and visual MC3TC adherence SEM images (×1000). (b–e) Fluorescence analysis. (f) Semiquantitative results of ALP. (g, h) ARS results and stereomicroscopic images of ARS-stained calcium deposits. *In vitro* investigations demonstrated that CAP-treated *n*HAP-biocomposites displayed enhanced osteogenic activity compared to untreated samples and were comparable to the positive control group (TCPs).

Immunofluorescence assay was used for determining the cell adhesion area (Figure 5a,b) and mean vinculin fluorescence intensity (Figure 5d). On average, the TCPs and H2@G20-P groups showed a larger area than the H2@G20 group at 6 h. Likewise, the H2@G20-P demonstrated significantly higher vinculin fluorescence intensity than H2@G20 at 3 and 6 h. An analysis of the vinculin intensity across the cell membrane using line scans shows that the relative fluorescence at 3 h was evenly distributed throughout the membrane (Figure 5c). Following 6 h of incubation, the relative fluorescence peaks of vinculin tended to concentrate in the center (Figure 5e).

We chose tissue culture polystyrene plastic (TCPs) as a positive control due to its conducive nature to cell adhesion^50^ and growth.^51^ Compared to the TCPs, CAP-treated *n*HAP-biocomposites had exceptional characteristics in terms of cell adhesion and proliferation. The reasons for this may be three-fold: First, the fillers within the *n*HAP-biocomposite became exposed (Figure 3g), leading to increased surface roughness^52^ and high hydrophilicity, both of which have been shown to favor cell adhesion.^53^ Second, the CAP device generated an abundance of hydroxyl ions.^54^ Chen et al. observed that, after exposure to plasmas, a decrease in carbon (%C) was observed, whereas oxygen (%O) increased, indicating the formation of new oxygen-containing polar moieties.^55^ Third, a mixture of GPs and *n*HAPs at a ratio of 10:1 (w/w) is used as fillers in our study; it is consistent with other research that plasma-treated *n*HAP or HAP is better at adhering and proliferative to cells.^56^ The optimization of filler blends could elicit variations in cellular adhesion and proliferation.^57^

Figure 5f plots the semiquantitative ALP activity of cells cultured on different substrates. The expression of ALP, an early osteogenic marker, markedly increased from 7 to 14 days of culture. A higher induction capability was demonstrated in the H2@G20-P (0.58 ± 0.12 nmol/min/mg) at day 14 compared to the H2@G20 group (0.45 ± 0.05 nmol/min/mg). The capability of *n*HAP-biocomposites to deposit minerals was assessed for 21 days. The absorbance of CAP-treated samples (2.90 ± 0.41) was higher than the non-CAP group (Figure 5g). As for the mineral-covered area, those calcium nodules appeared to stain more area in H2@G20-P (Figure 5h). Nodules of calcified bone matrix are the ultimate sign of terminal osteogenic differentiation. The preprocessed *n*HAP using CAP consisting of helium and oxygen could augment osteogenic activities and promote osteogenesis by our hypothesis.

**Figure 6:**
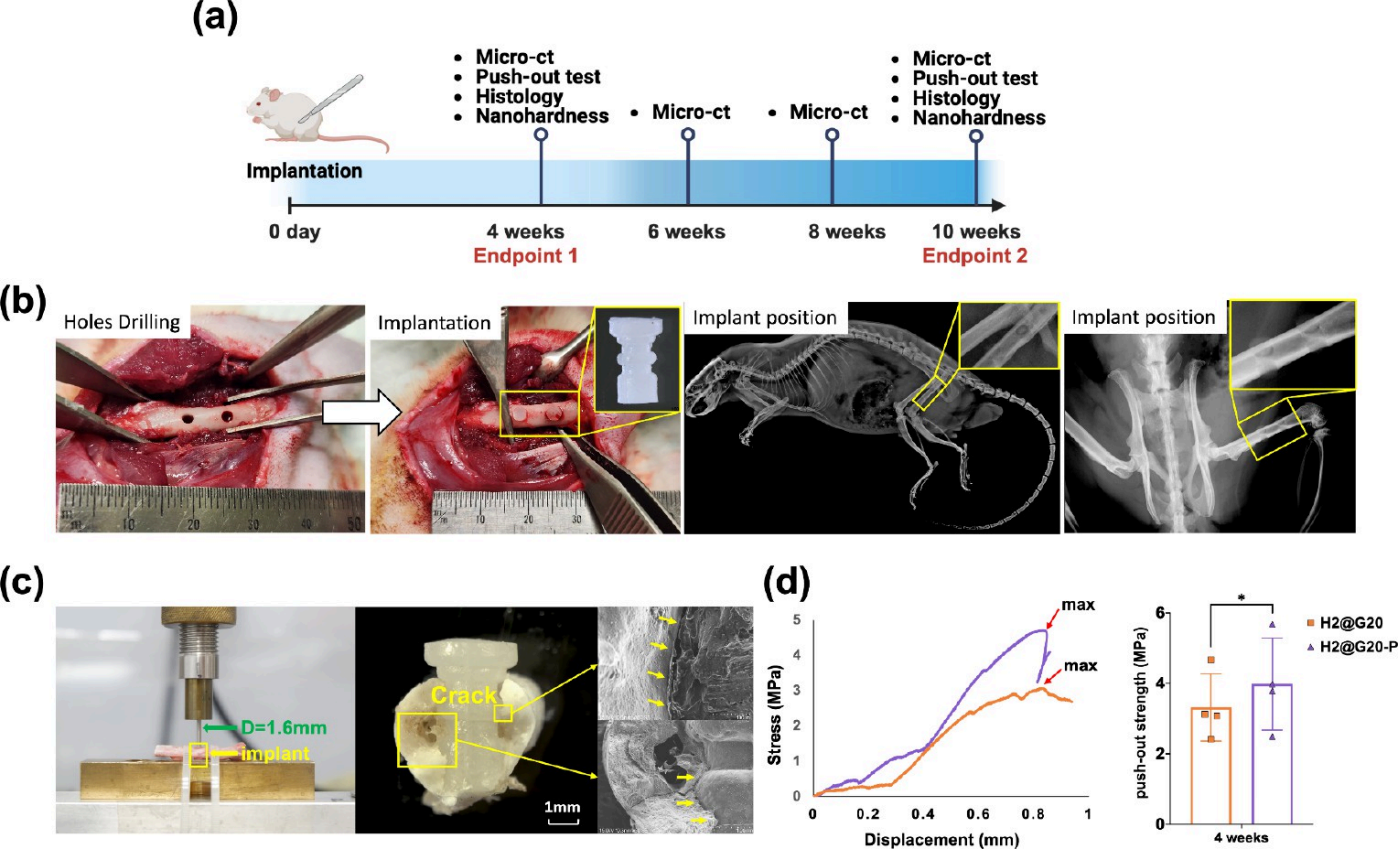
*In vivo* assessment procedure and bone-implant strength examination. (a) Surgery timeline and tests. (b) Surgery procedures and X-ray radiograph of the implant position. (c) Set-up diagram of bone-implant strength push-out test. (d) Representative stress–displacement curves and bone-implant strength push-out results for the 4 week group. In the 10 week group, all femurs fractured before the implant could be pushed out.

During the *in vivo* test, rats did not have lameness or infection following implantation surgery. Two groups of rats were euthanized after 4 and 10 weeks, respectively (Figure 6a,b). The 4 week post-operative PO test revealed that the *n*HAP-biocomposite implant had a strength of 3.08 (± 0.44) MPa, while the H2@G20-P implant had a higher strength of 3.86 (± 0.52) MPa. The representative stress–displacement curves are shown in Figure 6d, where the highest peak for the H2@G20-P surpassed that of the H2@G20 group. In the 10 week group, all femurs fractured before the implant could be pushed out, potentially due to the strong osseointegration between the irregular shape of the implant.

**Figure 7:**
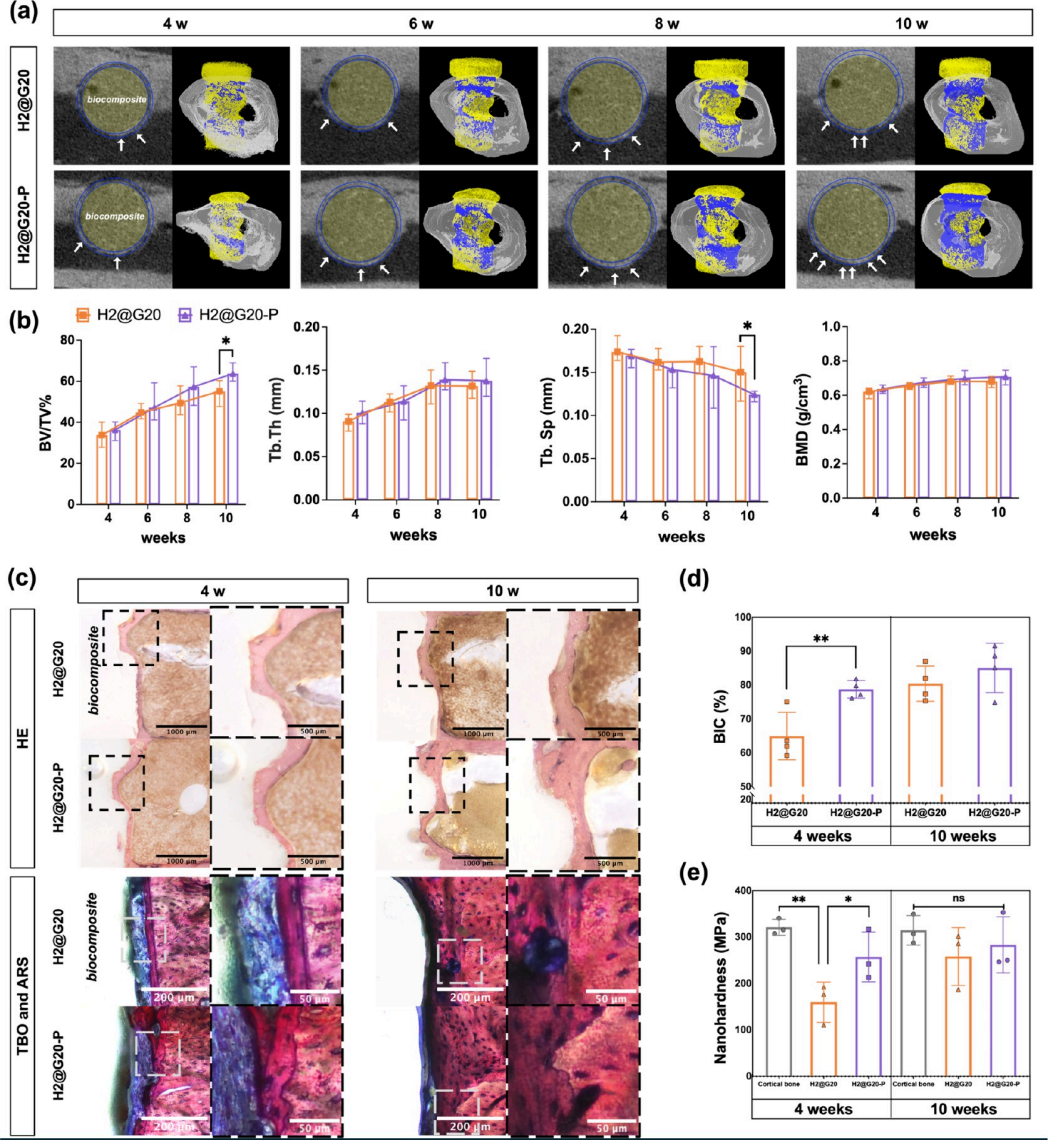
*In vivo* evaluation of the osseointegration performance of biocomposites in rat femur. (a) Representative 2D and 3D images of micro-CT reconstruction and (b) analytical results of trabecular bone volume to total volume fraction (BV/TV), trabecular thickness (Tb. Th), trabecular separation(Tb. Sp), and bone mineral density (BMD). (c) Histology staining for the implants and surrounding tissue. (d) Quantification of bone-implant contact ratio. (e) Measurement of nanohardness around implants compared to natural cortical bone.

Figure 7a shows the 2D and 3D results of micro-CT image reconstruction. It was evident that the bone deposition around the implant (yellow) was increasing in thickness (white arrow) and covering a broader area (blue). The BV/TV% results indicated a rise in the proportion of new bone tissue over time, with the H2@G20-P group demonstrating significantly higher results than the H2@G20 group at 10 weeks (Figure 7b). The distance between newly formed trabeculae decreased gradually over time, with the H2@G20-P group showing significantly lower results than the H2@G20 group at 10 weeks. Both trabecular bone thickness and bone mineral density exhibited an upward trend over time. Teotia et al.^58^ investigated a polymer composite containing a high proportion of Nano-HA and scaffolds. It was found that the composite can be used as a scaffold in critical bone defects (∼8 mm) as next generation synthetic bone substitutes.

New bony deposition was also evaluated by histological images (Figure 7c). As can be seen in the H&E slices, the new bone cytoplasm and extracellular matrix were stained shades of pink with eosin. The ARS staining distinguished new bone from old bone tissue, with new bones turning dark red and old bones turning light red. The TBO staining facilitated the visualization of fibrous encapsulation and covered the implant thread area. New bone tissue increased over time, with the H2@G20-P group exhibiting more. After 10 weeks, the boundary between the old and new bones gradually became blurred, and the new bones matured. The BIC results (Figure 7d) confirmed that at 4 weeks, the contact between the H2@G20-P and the implant was 64.97 ± 6.97%, which was significantly higher than that of the H2@G20 (78.78 ± 2.57%, *p* < 0.01). However, the difference gradually decreased after 10 weeks. The nanohardness of new bone tissue derived from plasma-treated implants was found to be statistically comparable to that of cortical bone. In contrast, the nanohardness of the non-CAP group was significantly lower than that of natural bone. However, after 10 weeks, the nanohardness of the non-CAP group increased. No statistical difference was observed between the experimental groups and cortical bone (Figure 7e).

On the basis of the interface reactions between the implant and the surrounding tissues, the *n*HAP-biocomposite implant should be classified as a biotolerant material, which is characterized by distance osteogenesis where the implant is surrounded by a fibrous connective tissue capsule,^59^ indicative of an immune response to the material. However, this kind of material has been successfully and widely used in orthopedic surgery despite fibrous encapsulation.^60^ Studies have yielded contradictory perspectives on the significance of the fibrous capsule on the surface of implant materials. Some researchers have suggested that the fibrous capsule isolates the body environment and minimizes adverse effects.^61^ Meanwhile, others propose that progressive thickening of the fibrous capsule may lead to excessive implant instability and failure.^62^ These processes are typically mediated by a series of protein adsorption events at the material surface and the development of an inflammatory environment, leading to fibrous capsules through crosstalk between immune cells and stromal cells (Figure 1).^63^ An appropriate next step would be investigating the immune response between *n*HAP-biocomposites and tissue.

Through CAP irradiation, OH− groups were introduced onto the material surface, resulting in a transition from a hydrophobic to a hydrophilic surface. The enhanced hydrophilicity of this surface facilitated the adsorption of proteins and polymers, which can have a positive impact on osteointegration.^64^ In addition, hydroxyapatite, which has a composition close to that of bone, has been proven to solve this problem.^65^ Due to its exceptional mechanical qualities^66^ and also its chemical and crystallographic similarity to natural apatite in bones,^67,68^ it serves as an excellent osteogenic differentiation promoter for bone cells.^69,70^ As mentioned above, the incorporation of *n*HAP into the matrix considerably improved the mechanical and osseointegration properties of the *n*HAP-biocomposite and rendered it a suitable candidate for medical implants at different anatomical sites. Its compatibility with 3D printing technology also rendered it appropriate for patient-specific scaffolds and cost-effective manufacture of complex geometries. This feature is particularly valuable in the reconstruction of irregularly shaped maxillofacial bones. The mechanical properties of the *n*HAP-biocomposite, which closely resemble those of natural bone, enable it to be applied in load-bearing environments such as spinal bone. Moreover, the CAP treatment-induced surface bioactivity also enhanced desirable osseointegration, particularly with the alveolar bone. Both *in vivo* and *in vitro* examinations confirm the material’s mechanical and biological functionality, justifying its viability for real-world clinical uses.

## 4 Conclusion

The *n*HAP-biocomposites exhibited excellent 3D printing manufacturability and high mechanical strength characteristics, such as collapse strength and compression modulus, comparable to those of PEEK, which are especially crucial for load-bearing applications. By subjecting polymer composites to cold plasma treatment, their surface hydrophilicity was enhanced by *n*HAPs being exposed on the outer surface, transforming the biotolerant surface into a bioactive one. This observation was substantiated by our *in vitro* experiments, where plasma-treated surfaces promoted cell adhesion and proliferation, evidenced by a stronger vinculin fluorescence intensity, increased expression of ALP, and higher deposition of calcium as indicated by ARS staining. *In vivo* testing has further confirmed the material’s remarkable bone integration properties. However, future research should comprehensively explore the mechanisms of osteogenesis and the immune response to live tissue.

## Supporting Information Available

Supplementary Animal Experimental Section; composition of resin-based *n*HAP-biocomposites materials; Teflon mold for *in vivo* resin-based biocomposite implant specimen; AFM surface topography of resin composite with different processing times; and EDX elemental maps of resin composite with different processing times (PDF)
Video S1: bone-implant bond strength test (MOV)

## Supplementary Material

am4c22032_si_001.pdf

am4c22032_si_002.mov
