# Supplementary material for: Engineered 3D-Printable Nanohydroxyapatite Biocomposites with Cold Plasma-Tailored Surface Features to Boost Osseointegration
Source: ACS Appl Mater Interfaces. 2025 Apr 14;17(16):23522–35. doi: 10.1021/acsami.4c22032 (PMC12022952; doi:10.1021/acsami.4c22032)
Supplement: Supplementary file 1 — am4c22032_si_001.pdf [file am4c22032_si_001.pdf]

Supporting Information

Engineered 3D-Printable Nanohydroxyapatite Biocomposites with Cold Plasma-Tailored Surface Features to Boost Osseointegration

Rosalind Sin Man Chan [a], Sang Jin Lee [a], Fang Wang [b], Tianyu Zhou [a], Ravi Kishan [c], Ho Cheung Shum [b,d], Weifa Yang [c], Yu-xiong Su [c], James Kit Hon Tsoi [a], Ashish D. Diwan [e,f], B. Gangadhara Prusty [g,h], Kiho Cho [a,g,]*

[a] *Division of Applied Oral Sciences and Community Dental Care, Faculty of Dentistry, The University of Hong Kong, Hong Kong SAR 999077, China*

[b] *Department of Mechanical Engineering, The University of Hong Kong, Hong Kong SAR 999077, China*

[c] *Division of Oral and Maxillofacial Surgery, Faculty of Dentistry, The University of Hong Kong, Hong Kong SAR 999077, China*

[d] *Advanced Biomedical Instrumentation Centre, Hong Kong Science Park, Shatin, New Territories, Hong Kong SAR 999077, China*

[e] *Spine Labs, St George and Sutherland Clinical School, University of New South Wales, Randwick, NSW 2052, Australia*

[f] *Spine Service, Department of Orthopaedic Surgery, St George and Sutherland Clinical School, University of New South Wales, Kogarah, NSW 2217, Australia*

[g] *School of Mechanical and Manufacturing Engineering, University of New South Wales, NSW 2052, Australia*

[h] *ARC Centre for Automated Manufacture of Advanced Composites, School of Mechanical and Manufacturing Engineering, University of New South Wales, Sydney, NSW 2052, Australia*

[*] *Corresponding authors: K. Cho (dkcho@hku.hk)*

Supplementary Animal Experimental Section

Animal holding methods: These animals were sourced from Charles River Lab (USA) and bred under an AAALAC International accredited program at the Centre for Comparative Medicine Research, The University of Hong Kong (HKU), under Specific Pathogen Free (SPF) conditions. The rats were housed individually in ventilated cages within environmentally controlled rooms, with a 12:12 dark-light cycle. They were provided *ad libitum* with a laboratory diet manufactured by LabDiet (USA).

***In vivo* surgery procedures:** Under sterile conditions, the rats were anesthetized with a mixture of Ketamine (100 mg/kg) and Xylazine (10 mg/kg) via intraperitoneal injection. The rats were placed in the lateral decubitus position, and the left thigh was shaved and disinfected with chlorhexidine gluconate 2% w/v and 70% ethanol. An incision with 5 cm long was made along the axis of the tibia. The tibia was exposed by carefully blunt dissection of the mucosa and muscles. A dental surgical motor (SM3, OSSTEM, South Korea) was employed to drill holes at a speed of 1500 min^{-1} with a torque of 50 N·cm. Initial drilling was performed using a lance drill (D=2.00 mm), followed by hole expansion with a twist drill (D=2.2 mm) with continuous sterile saline used for cooling. The distance between the two holes was approximately 5 mm. Following implantation, the muscle and skin layers were individually sutured with resorbable sutures (Vicryl 4.0, Ethicon Product) and staples, respectively. Intraoperative monitoring was conducted every 15 minutes. Post-operative recovery included analgesia and daily monitoring of healing and mobility.

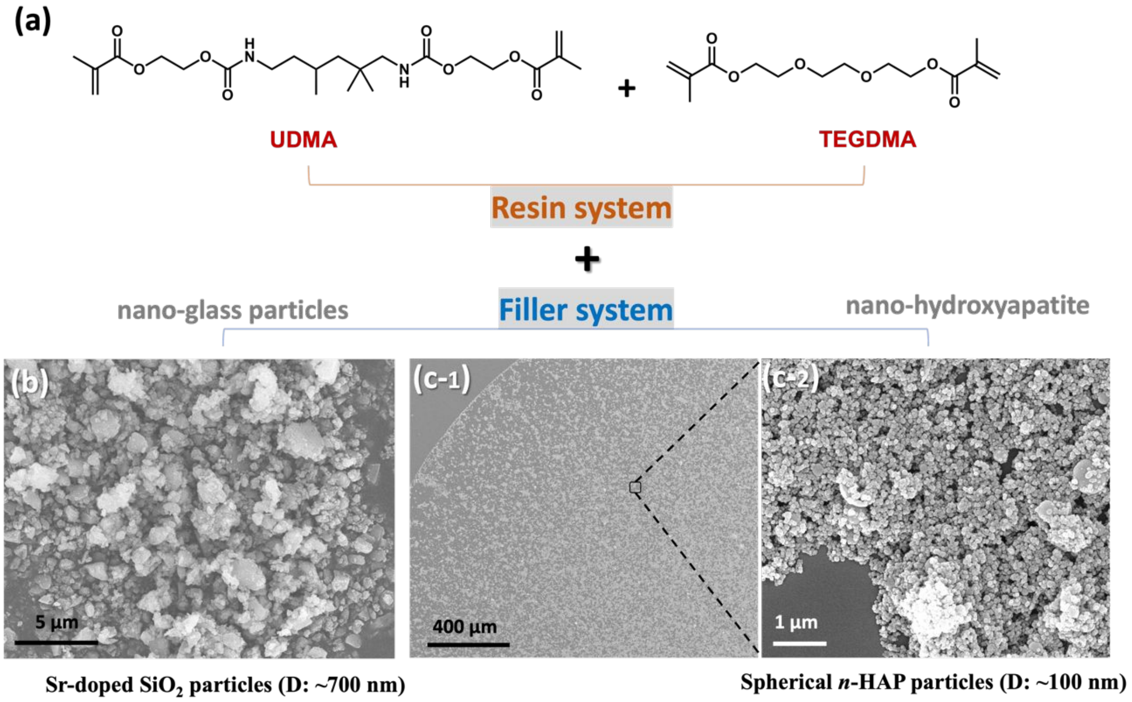

Figure S1. Composition of resin-based nHAP-biocomposites materials. 3D-printable biocomposites formulated with a combination of **(a)** UDMA and TEGDMA, **(b)** SEM image of strontium-doped SiO_2 glass particles, **(c-1)** Low magnification SEM image of *n*HAP particles, and **(c-2)** high magnification *n*HAP particles image from the area marked in **(c-1)**.

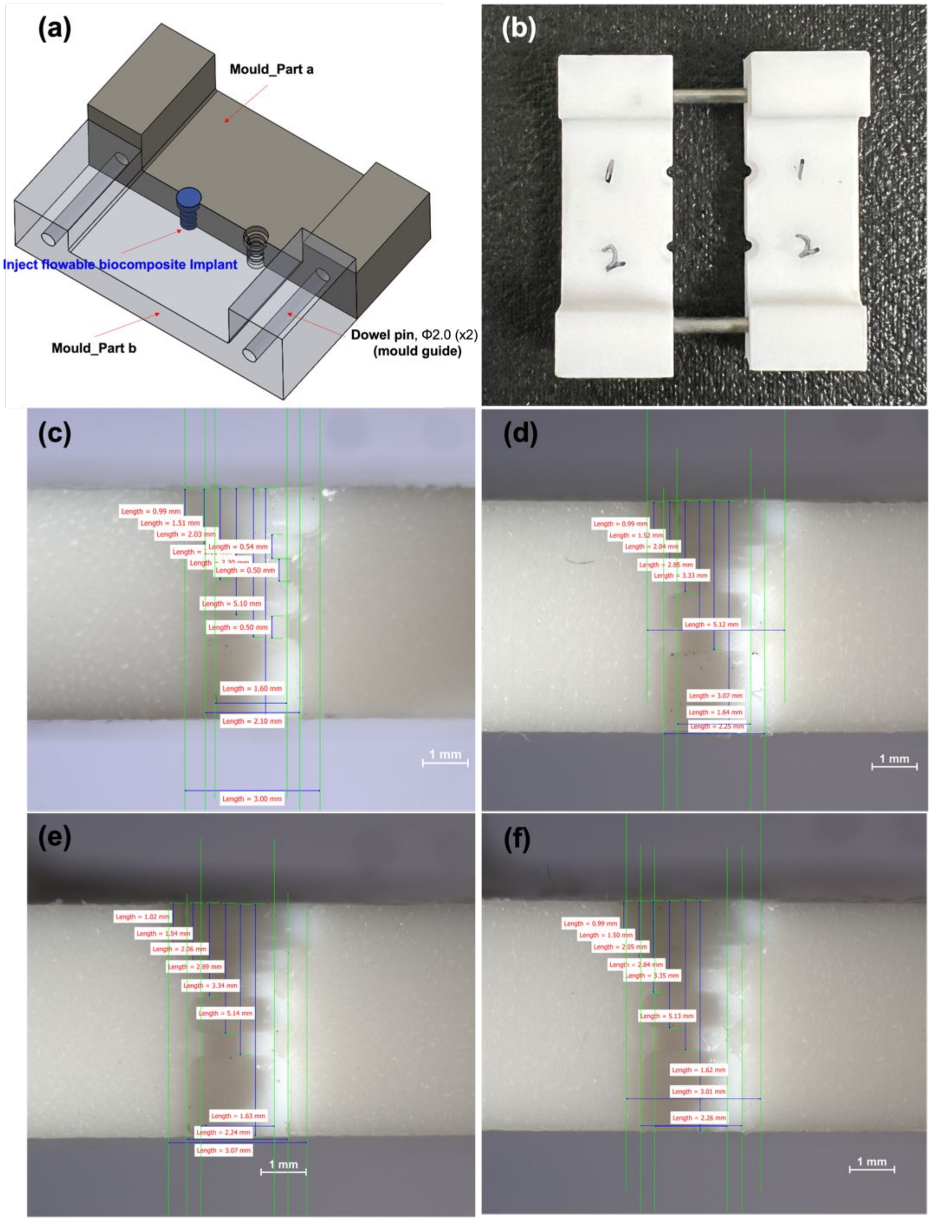

Figure S2. Teflon mould for *in vivo* resin-based biocomposites implant specimen. **(a)** Schematic of the Teflon mould design for mini-implant in *in vivo* study. **(b)** Photo of Teflon mould. Dowel pins secure the two halves of the mould. Flowable HA-composite was injected into the hole and cured by a LED light curing unit. **(c)-(f)** Sagittal plane of Teflon mould.

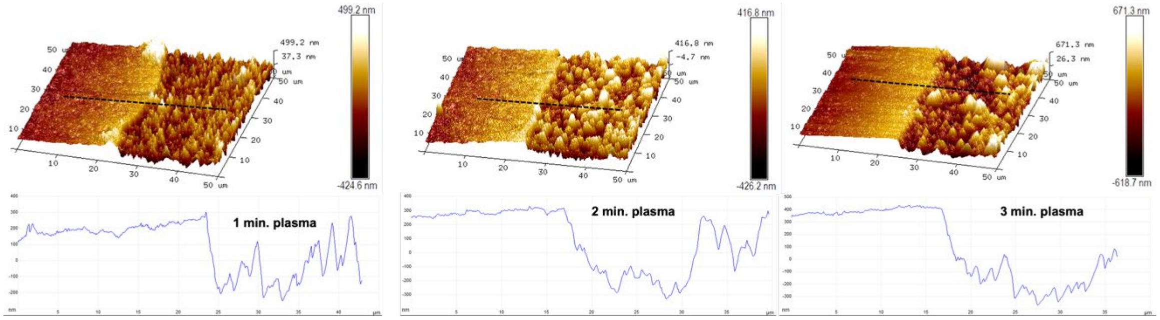

Figure S3. AFM surface topography of resin composite with different time processing.

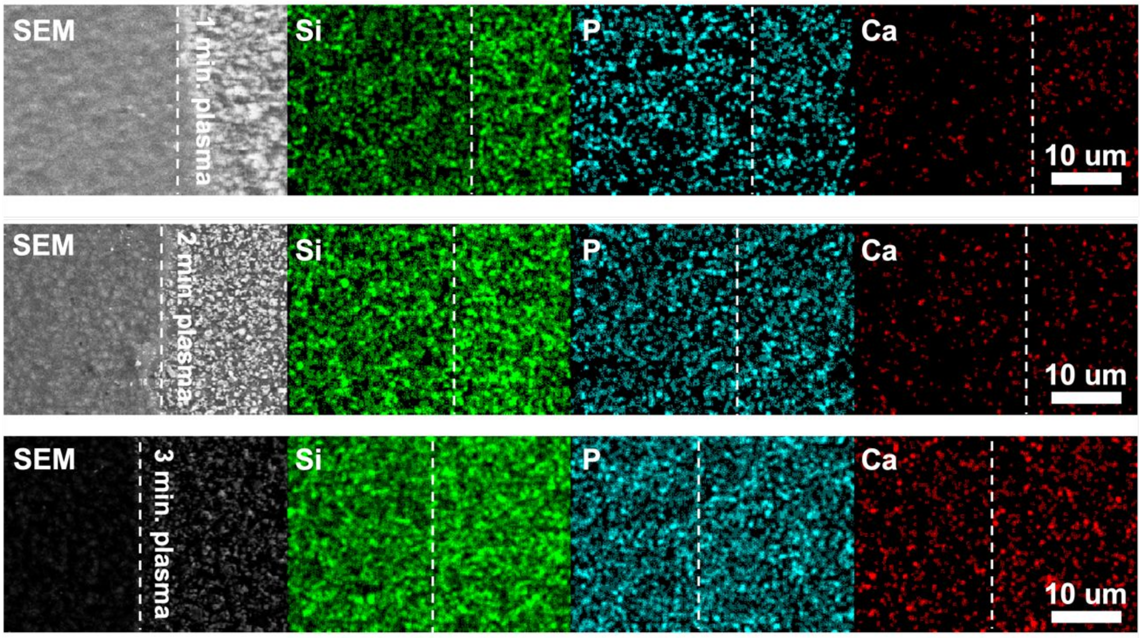

Figure S4. Elemental maps of EDX spectrum of resin composite with different time processing. These maps show mild changes in the surface distribution of silicon (Si), phosphorus (P), and calcium (Ca).

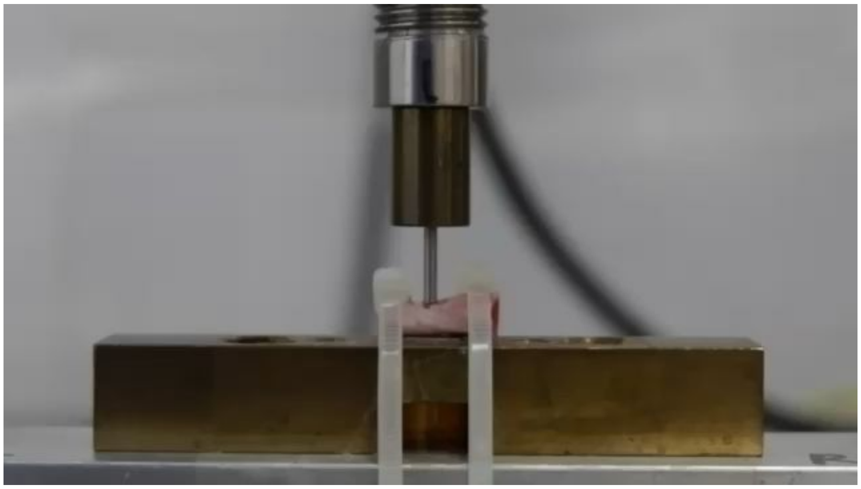

Video S1. Video of bone-implant bond strength test.
